# An all-in-one, Tet-On 3G inducible PiggyBac system for human pluripotent stem cells and derivatives

**DOI:** 10.1038/s41598-017-01684-6

**Published:** 2017-05-08

**Authors:** Lauren N. Randolph, Xiaoping Bao, Chikai Zhou, Xiaojun Lian

**Affiliations:** 1 Department of Biomedical Engineering, Pennsylvania State University, University Park, PA 16802 USA; 2 Department of Biology, Pennsylvania State University, University Park, PA 16802 USA; 3 The Huck Institutes of the Life Sciences, Pennsylvania State University, University Park, PA 16802 USA; 4 Department of Chemical and Biological Engineering, University of Wisconsin, Madison, WI 53706 USA; 5 Department of Cell and Molecular Biology, Karolinska Institutet, 17177 Stockholm, Sweden

## Abstract

Human pluripotent stem cells (hPSCs) offer tremendous promise in tissue engineering and cell-based therapies due to their unique combination of two properties: pluripotency and unlimited proliferative capacity. However, directed differentiation of hPSCs to clinically relevant cell lineages is needed to achieve the goal of hPSC-based therapies. This requires a deep understanding of how cell signaling pathways converge on the nucleus to control differentiation and the ability to dissect gene function in a temporal manner. Here, we report the use of the PiggyBac transposon and a Tet-On 3G drug-inducible gene expression system to achieve versatile inducible gene expression in hPSC lines. Our new system, XLone, offers improvement over previous Tet-On systems with significantly reduced background expression and increased sensitivity to doxycycline. Transgene expression in hPSCs is tightly regulated in response to doxycycline treatment. In addition, the PiggyBac elements in our XLone construct provide a rapid and efficient strategy for generating stable transgenic hPSCs. Our inducible gene expression PiggyBac transposon system should facilitate the study of gene function and directed differentiation in human stem cells.

## Introduction

Human pluripotent stem cells (hPSCs) can be propagated indefinitely while still retaining the capacity to differentiate into all somatic cell types^1, 2^. This infinite cell source is of great interest for probing cellular differentiation processes with the goal of creating cell-based therapies for a range of degenerative diseases. In order to achieve clinically viable cell therapies, new technologies are needed to facilitate a deeper understanding of how transcription factors temporally regulate stem cell differentiation. For example, engineering hPSCs to increase or reduce expression of a specific gene would provide a useful way to decode the gene’s role in complex cell signaling networks, as well as its function in stem cell differentiation. hPSCs are one of the most challenging cell types to genetically engineer due to the low transfection efficiencies and promoter-dependent silencing during differentiation^3^. Temporally changing gene expression patterns as the stem cells differentiate represents a key milestone in hPSC genetic engineering. This would further unlock the potential of hPSC technology advancing the understanding of human development and disease to support clinical treatment advances.

Treatment of various degenerative disorders using stem cell therapies requires directed differentiation of hPSCs into clinically applicable cell types. Many *in vitro* directed differentiation protocols and methods rely on mimicking *in vivo* animal embryonic development by providing cells with stage-specific stimuli, including growth factors and small molecules, to modulate cell signaling pathway activity^4^. For example, cardiomyocyte differentiation requires precise and sequential activation and inhibition of the Wnt/β-catenin pathway^5, 6^. Pancreatic β cell differentiation necessitates application of stage-specific soluble inductive signals for differentiation of hPSCs to definitive endoderm, pancreatic progenitor, endocrine progenitor, and the terminally differentiated β cell state^7^. The temporal dependence of differentiation processes makes them unique and demands genetic engineering tools capable of dissecting and manipulating these cellular events.

Plasmid constructs are often used to interrogate the function of specific cellular genetic elements. Many plasmids use a constitutive promoter to express a gene of interest. While these plasmids are useful for some applications where gene expression is continuously required, they are not suitable for human stem cell differentiation applications where temporal control of gene expression is crucial. Inducible plasmid constructs are more effective for stem cell differentiation applications due to increased user control of the gene expression. Incorporation of a drug inducible promoter is one design strategy used to achieve an inducible plasmid with tight temporal regulation. Drug inducible promoters that rely on drug activation mechanisms, as opposed to suppression mechanisms, improve user manipulation of a gene’s temporal expression kinetics^8^. The Tet-On 3G system employs a doxycycline-binding transactivator protein and a low background promoter to regulate gene transcription. The expression level of a gene of interest under the pTRE3G promoter can be modulated by changes in doxycycline (Dox) concentration^8, 9^.

Plasmid systems implementing transposon technology provide an advantage by allowing reversible insertion and removal from the genome. The PiggyBac transposon is an example of an element that can transpose genetic cargo, including larger DNA sequences, into the human genome with higher transposition activity than commonly used transposons such as hyperactive Sleeping Beauty^10, 11^. While random plasmid integration lacks specificity for an integration site, it provides the advantage of a rapid and efficient means of generating stable hPSC gene expression. In addition, PiggyBac based systems generate multiple integration sites within the human genome, which can reduce the likelihood of the construct being silenced. This application of multiple integration sites aims to resolve the current issue of engineered gene control deterioration due to construct silencing during human stem cell differentiation. The Tet-On 3G system has been used in a lentiviral system and with a safe harbor site knock-in approach using TALEN genome editing technology^12^. However, both of these integration strategies require more time, and in the case of the knock-in approach, efficiency is notably lower.

Here we present a novel plasmid that combines the PiggyBac transposon and Tet-On 3G promoter elements providing tight user control of temporal and tunable gene expression. Kinetic characterization of our XLone inducible gene expression system demonstrates sensitive gene regulation can be achieved in undifferentiated pluripotent stem cells and in terminally differentiated cells using our system.

## Results

### Establishment of inducible GFP expression in hPSCs via the XLone plasmid

**Figure 1:**
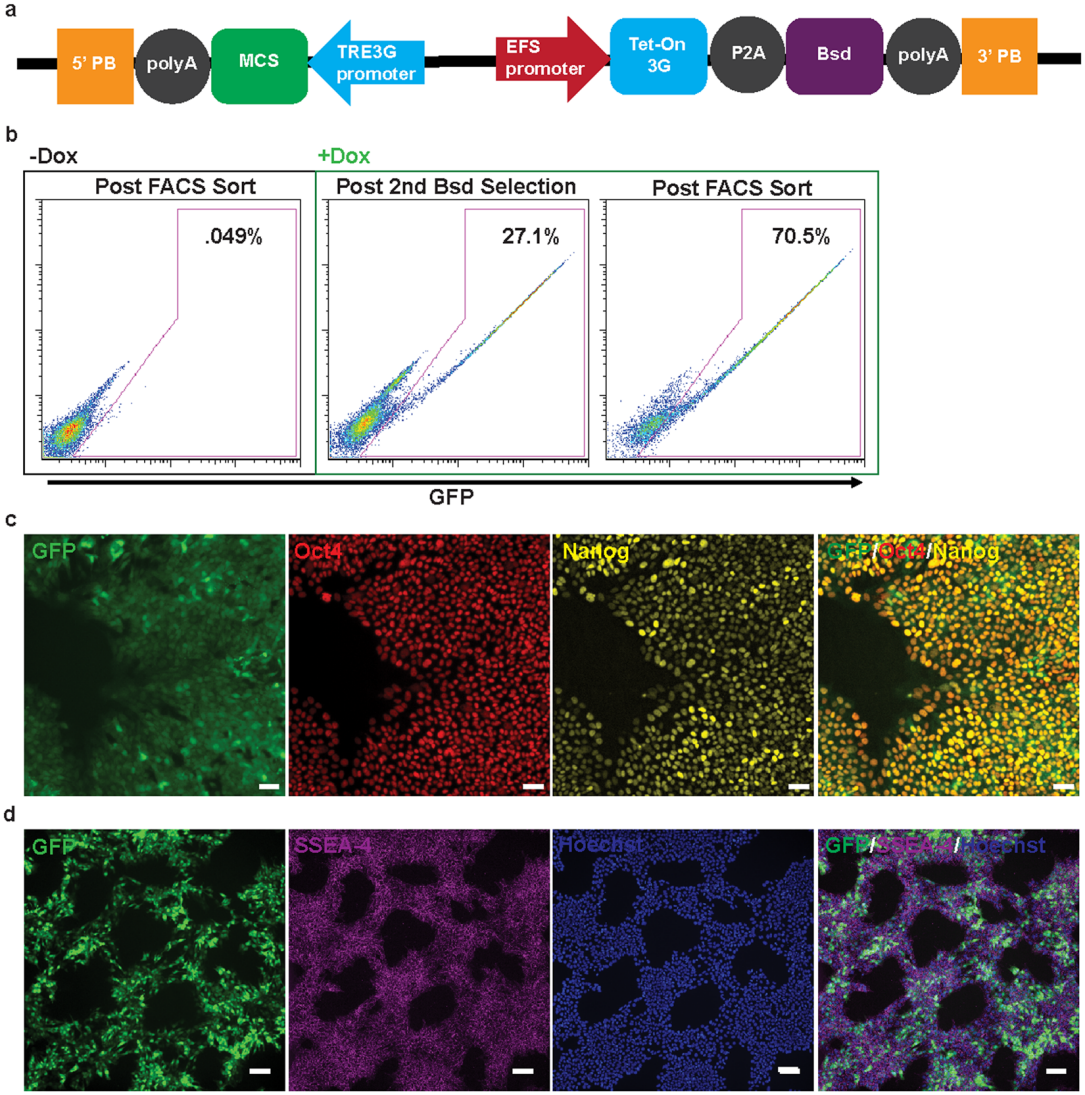
Design and generation of XLone transgenic hPSCs. (**a**) Schematic showing transposable plasmid cassette design. The ColE1 origin of replication and ampicillin resistance gene for *E.coli* amplification are not shown. 5′ PB, 5′ PiggyBac Terminal Repeat; 3’ PB, 3’ PiggyBac Terminal Repeat; MCS, Multiple Cloning Sites; Bsdr, Blasticidin resistance gene. (**b**) The tight control of gene expression (GFP) is shown to have less than 1% GFP positive cells without the addition of Dox. Flow cytometry analysis of the cells after Bsd drug selection and FACS live cell sort. Live cell sorting enriched the population to maximal 70.5% GFP positive. (**c**) GFP positive cells with immunostaining of transcription factor pluripotency markers Oct4 and Nanog. Scale bars are 50 µm. (**d**) GFP positive cells with immunostaining of cell surface pluripotency marker SSEA-4. Scale bars are 100 µm.

**Figure 2:**
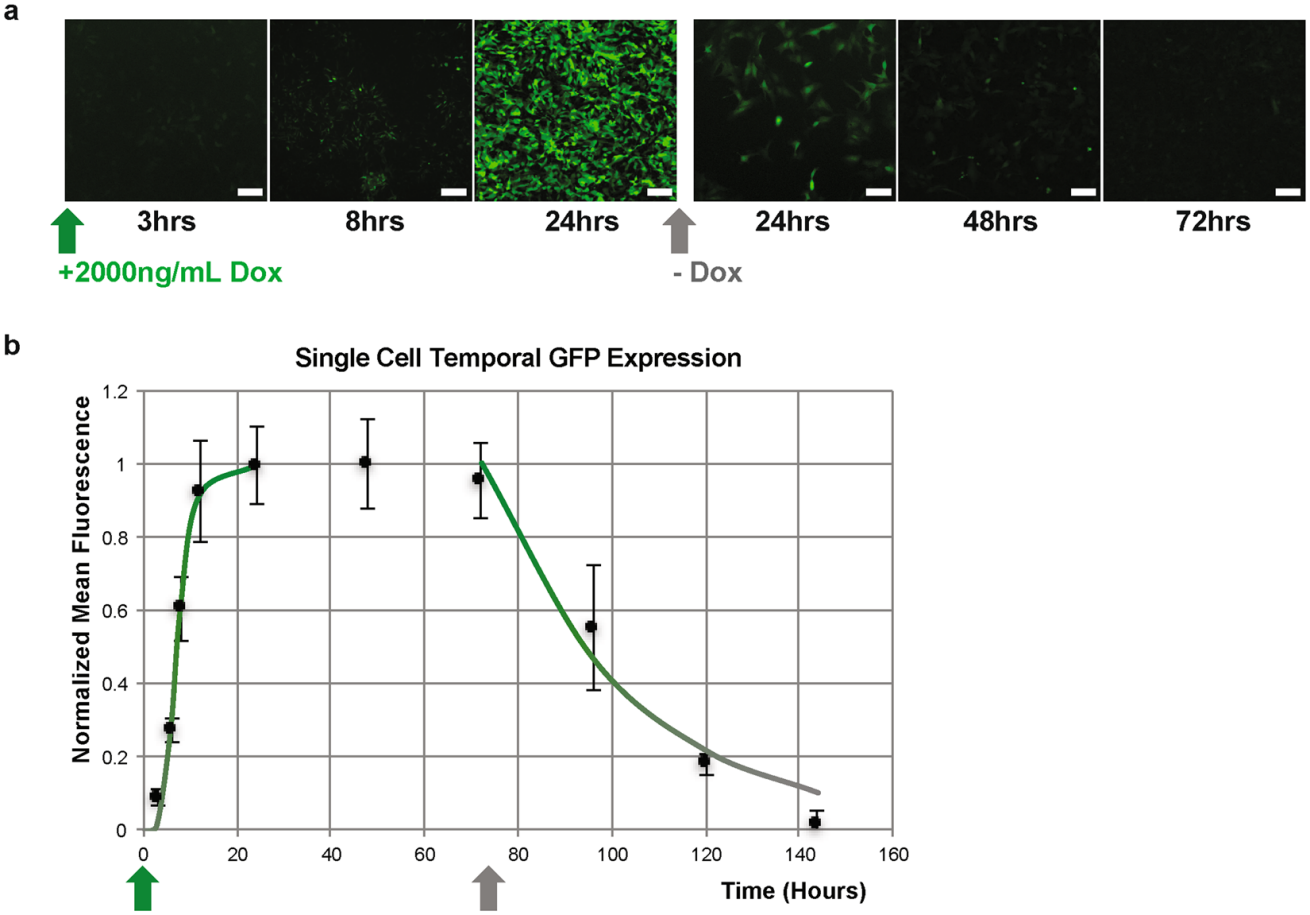
Characterization of XLone temporal expression pattern. (**a**) Fluorescence microscopy images showing the change in GFP expression over time with and without doxycycline. Scale bars are 100 µm. (**b**) Plot illustrating the normalized average corrected total fluorescence of a single cell over time as GFP expression turns on, reaches a steady state maximum, and turns off. The non-linear curve fits are described by the on and off rate equations and the error bars show standard error of the mean.

Our newly designed plasmid, **XLone**, incorporates flanking PiggyBac inverted terminal repeats, two promoters and corresponding poly(A) sequences (Fig. 1a). The first promoter, the TRE3G promoter, controls the expression of elements inserted into the multiple cloning site (MCS) located downstream of the TRE3G promoter. The second promoter, a constitutive EF1α promoter, controls expression of a resistance gene for drug blasticidin (Bsd) and the Tet-On 3G transactivator protein (Fig. 1a). We chemically synthesized all the necessary DNA elements and assembled them into the pUC57 backbone with an ampicillin resistance gene for bacteria selection. We then cloned a GFP gene into the MCS of the XLone plasmid to generate an XLone-GFP plasmid. This resulting plasmid construct was then transformed into *E.coli* for amplification and purified with a plasmid purification kit. We co-transfected our plasmid XLone-GFP and a pCYL43 plasmid^13^ containing PiggyBac transposase into human embryonic stem cells (H9 line). Two rounds of one-day Bsd drug selection enriched the population of cells with the plasmid incorporated to 27.1% (Fig. 1b). FACS sorting yielded more GFP positive cells as compared to the two rounds of one-day Bsd drug selection (Supplementary Fig. 1 and Fig. 2). Therefore, we decided to use FACS live sorting method for the purification of the cells responsible for the Dox inducible GFP expression. Dox was administered to the cells at 2000 ng/mL and live cell FACS sorting was performed to isolate a pure GFP positive population of cells (Fig. 1b). The purified GFP positive cells were maintained in culture without Dox for further characterization experiments. After several months (>3 months) in culture, the cells incorporated with XLone-GFP construct were treated with Dox for 24 hours. The cells were then paraformaldehyde fixed and immunostained with pluripotency markers Oct4, Nanog, and SSEA-4. Immunostaining experiments showed the transgenic cells retained pluripotency as well as inducible GFP expression (Fig. 1c and d).

### Temporal gene expression is characterized by the Hill equation

To characterize the temporal kinetics of GFP expression, Dox was administered to the cells at a 2000 ng/mL dose. Cells were then imaged 3, 6, 8, 12, 24, 48, and 72 hours after dosing. After 72 hours, Dox was removed from the system, and the cells were passaged. The cells were then imaged every 24 hours until no GFP expression was observed. The images from each time point were analyzed, and the average corrected total fluorescence from a single cell was calculated and normalized using ImageJ software (Fig. 2).

1 $$Kinetic\,\,On\,Rate=\frac{{t}^{h}}{{t}^{h}+{{\tau }_{on}}^{h}}$$

2 $$Kinetic\,\,Off\,\,Rate={e}^{-t/{\tau }_{off}}$$

The expression on rate can be described by the Hill equation using temporal variables where τ_on_ is the time to reach half maximal expression (Eq. 1)^14, 15^. The Hill coefficient was found to be 4.7, and the τ_on_ value was fitted at 7.3 hours. This demonstrates that the XLone system is able to achieve half of the total expression expected for a given Dox concentration within 7.3 hours and full expression within approximately 14 hours. The rapid on rate for gene expression via XLone proves relevance for hPSC differentiations that require a 24-hour average period for signaling pathways activation and downstream gene expression. Maximal expression is maintained by continuous dosing with Dox. Upon Dox removal from the system, the expression decays exponentially with dependence on τ_off_, which is 31.3 hours for this system based on non-linear curve fitting and least squares analysis (Eqn. 2)^14, 15^. The gene expression half-life, τ_off_, indicates that expression rapidly decreases with the removal of Dox in a time period applicable to the temporal modulations used in hPSC differentiation.

### Tunable gene expression is characterized by Michaelis-Menten kinetics

**Figure 3:**
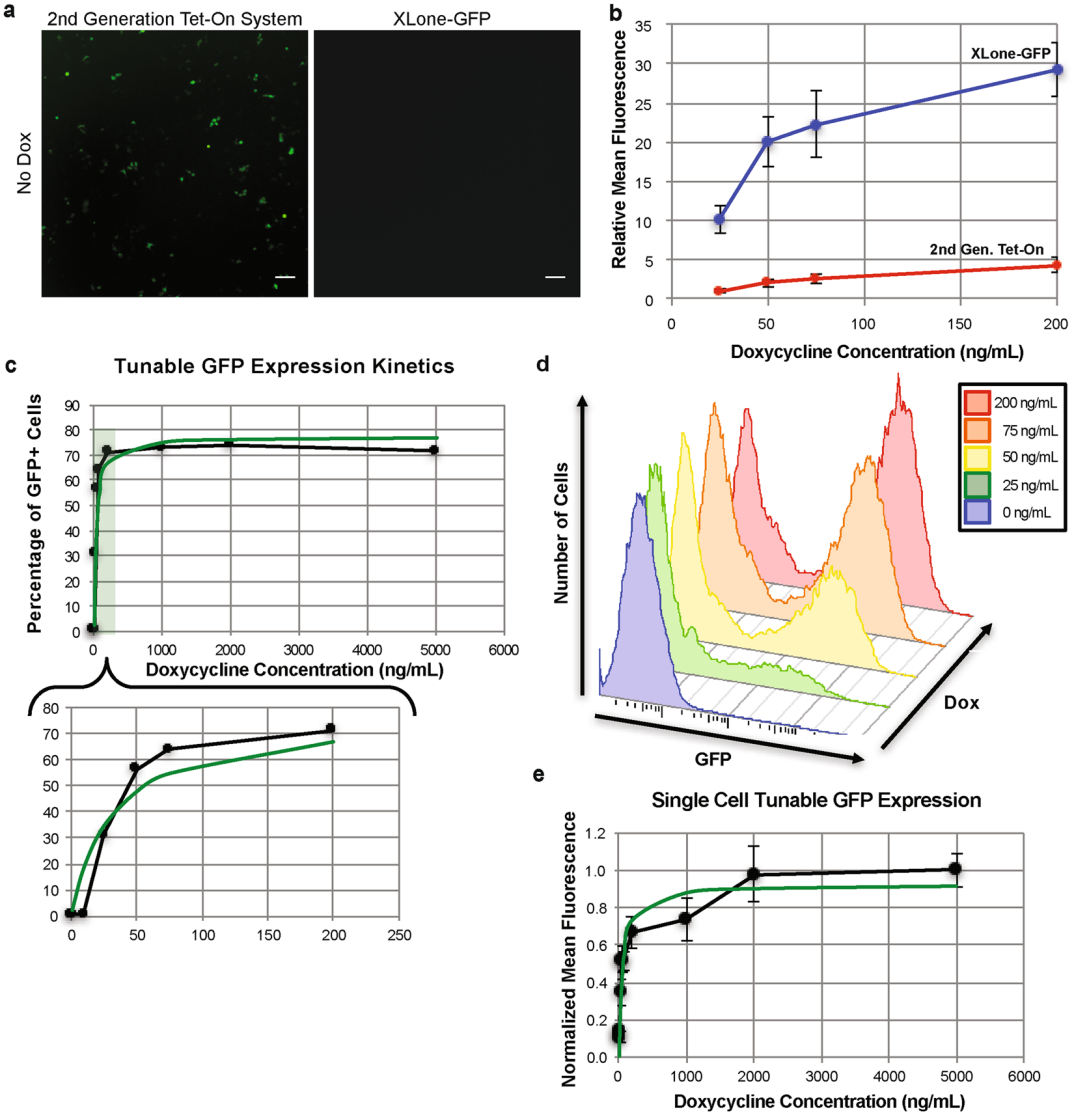
Characterization of XLone tunable expression pattern. (**a**) Fluorescence microscopy images showing background (without Dox exposure) GFP expression comparison between a 2^nd^ generation Tet-On system and the XLone-GFP construct in 293TN cells. Scale bars are 100 µm. (**b**) Plot showing the mean fluorescence of 293TN cells exposed to lower Dox concentrations. The blue and red lines represent the XLone-GFP construct and 2^nd^ generation Tet-On system respectively. Error bars show standard error of the mean (n = 18). Student t-test was used for statistical analysis for each Dox concentration comparing the 2^nd^ Tet-On system and XLone system. (**c**) Tunable expression kinetics of the inducible plasmid system fit by the Michaelis-Menton equation with K_m_ of 30.8 ng/mL and V_max_ of 77.32%. (**d**) Flow cytometric analysis of tunable GFP expression. The plot shows changes in the number of cells expressing GFP with increasing doxycycline concentration over the 10–200 ng/mL dosing range. (**e**) Plot illustrating the normalized average corrected total fluorescence of single cells with exposure to varied doxycycline concentrations. The non-linear curve fit is based on Michaelis-Menten kinetics with a fitted K_m_ of 51.2 ng/mL and V_max_ of 0.92. The error bars shown represent the standard error of the mean.

Besides the ability to temporally modulate gene expression in hPSCs, we are also interested in precise control of the amount of gene expression. We first compared the background leakage expression of GFP of the XLone-GFP construct to a 2^nd^ generation Tet-On system. After transfection of both plasmid systems into 293TN cells, in the absence of Dox treatment, we observed notable background expression of GFP in 293TN cells transfected with the 2^nd^ generation Tet-On system (Fig. 3a). The cells transfected with the XLone-GFP construct showed no visible GFP expression in the absence of Dox, demonstrating the reduced background expression in XLone as compared to the 2^nd^ generation Tet-On system (Fig. 3a). We then compared the sensitivities of the two systems at low Dox concentrations. Increased GFP expression was observed over the 25–200 ng/mL Dox concentration range for the XLone-GFP construct. The difference in GFP expression level between the two plasmid constructs was found to be statistically significant across the 25–200 ng/mL Dox concentration range by student t-test (p < 0.005) (Fig. 3b). To further characterize the tunable expression feature of our XLone system, transgenic hPSCs with the XLone-GFP were dosed with various concentrations of Dox: 1, 10, 25, 50, 75, 200, 1000, 2000, and 5000 ng/mL. After 24 hours of incubation with Dox, the cells were imaged, dissociated, and analyzed via flow cytometry to determine the percent of the population expressing GFP (Fig. 3c and d). The images for each Dox concentration were analyzed and the average corrected total fluorescence from a single cell was calculated and normalized using ImageJ software (Fig. 3e). The gene expression level showed increased sensitivity for Dox concentrations between 10 and 200 ng/mL with maximal expression across the population achieved at concentrations higher than 200 ng/mL (Fig. 3c). The tunable expression data, for both population and single cell characterizations, are best modeled by Michaelis-Menten kinetics with K_m_ values of 30.8 ng/mL and 51.2 ng/mL and V_max_ values at 77.32% and 0.92 respectively^16, 17^. This demonstrates tunable gene expression control on both single cell and multi-cellular scales. The small K_m_ values indicate the system has high sensitivity even for low Dox concentrations; this is advantageous for achieving precise control of gene expression level. When translating this technology to an *in vivo* system, a higher dose of Dox can lead to undesirable side effects, and longer clearance time, and thus prolonged gene expression following Dox withdrawal, highlighting the importance of our system’s small K_m_ value^18^.

### Transgene is inducible in hPSC-derived cardiomyocytes

**Figure 4:**
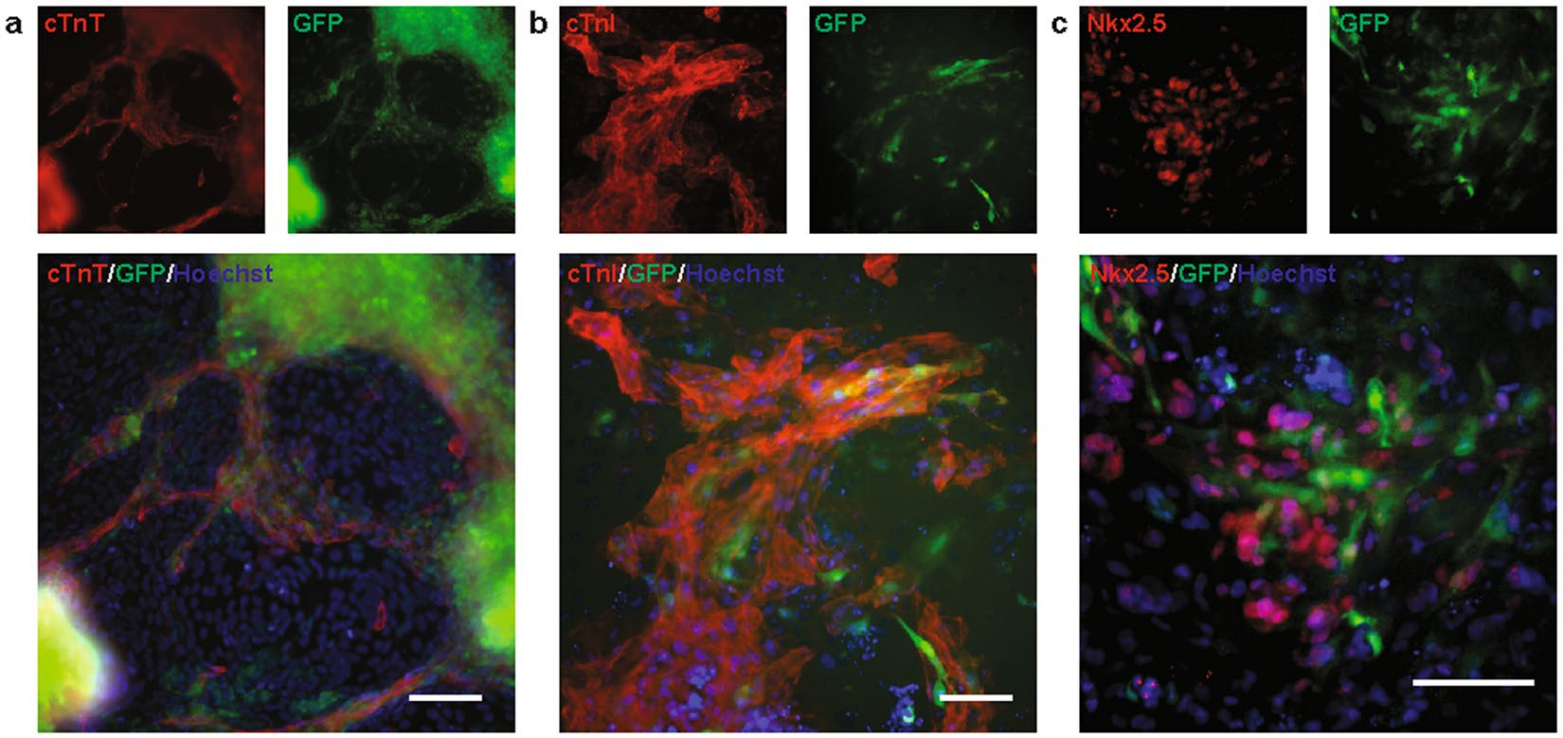
Characterization of GFP expression in differentiated cells. Immuno-fluorescence imaging showing the co-localization of GFP expression and cardiomyocyte markers cTnT (**a**), cTnI (**b**), and Nkx2.5 (**c**) in hPSC derived cardiomyocytes. Scale bars are 100 µm.

Transgenic hPSCs often silence foreign gene expression during human stem cell differentiation. To test whether our XLone transgene construct sustains expression in stem cell differentiated cells, we differentiated the transgenic hPSCs into cardiomyocytes using the GiWi protocol^5^. After the differentiated cells exhibited spontaneous contraction, the beating cardiomyocytes were incubated with 2000 ng/mL Dox for 24 hours. The cells were then fixed and immunostained for various cardiac markers including cardiac troponin T (cTnT), cardiac troponin I (cTnI), and cardiac transcription factor Nkx2.5. Fluorescence microscopy showed the differentiated cardiomyocytes were GFP positive (Fig. 4), indicating the gene construct was not silenced during differentiation.

## Discussion

We designed, synthesized, and characterized an inducible plasmid system, XLone, which combines the PiggyBac transposon and Tet-On 3G promoter elements to achieve precise temporal and tunable control of gene expression. Our plasmid was successfully integrated into hPSCs, and the transgenic cell population could be easily enriched via drug selection or FACS sorting. Furthermore, our inducible plasmid system exhibited negligible background expression. After several months in culture, the hPSCs maintained inducible GFP expression and were positive for nuclear pluripotency markers Oct4 and Nanog as well as cell surface pluripotency marker SSEA-4.

The temporal expression profile can be described by the Hill equation, with a Hill coefficient of 4.7 indicating positive cooperative binding^19^. This is consistent with the expected behavior of the Tet-On 3G system where Dox binds first to the transactivator protein enabling it to bind to the TRE3G promoter and drive transcription. Half maximal expression was predicted to occur 7.3 hours after Dox administration, and full expression was observed between 12 and 24 hours post stimulation. Expression decayed exponentially with the removal of Dox with a predicted half-life of 31.3 hours.

Tunable expression of the XLone plasmid system followed Michaelis-Menten kinetics on both single cell and multi-cellular scales with K_m_ doxycycline concentrations of 30 ng/mL and 51.2 ng/mL. Both modeled parameters fall in the observed range of increased Dox sensitivity between 10–200 ng/mL. Previous generations of the Tet-On system, such as the first generation Tet-On system, displayed a much lower sensitivity to Dox, acting in the 100–500 ng/mL range^18^. Additionally, we demonstrated the XLone-GFP plasmid had much lower background GFP expression and increased GFP expression when exposed to lower Dox concentrations as compared to the 2^nd^ generation Tet-On system.

After long-term culture of the transgenic hPSCs in stem cell media, the percentage of GFP expression of our FACS purified cell population was observed to reach a maximum at 77.8%. While theoretically we would expect to observe a 100% positive population, we attribute the observed reduction to silencing of the integrated construct after long-term culture of hPSCs. Our plasmid system showed continued inducible expression after differentiation to a terminally differentiated somatic cell type as evidenced by co-localization of GFP expression and cardiac marker (cTnT, cTnI, and Nkx2.5) immunostaining.

Our XLone plasmid design will allow future studies to closely examine the temporal regulation of gene function in development and in clinically relevant differentiated cell types with tunable expression control. This technology permits further research aimed at understanding gene expression during human stem cell differentiation and how cellular signals direct cell fate and differentiation decisions. The use of this plasmid system to elucidate and study directed differentiation to disease relevant cell types would propel stem cell research closer to clinical application.

## Methods

### Maintenance of hPSCs

All cell culture experiments involving human cell lines were approved by the Embryonic Stem Cell Oversight Committee at the Pennsylvania State University and carried out in accordance with the approved guidelines. Informed consent was obtained from all subjects. Human pluripotent stem cells (H9) were maintained on Matrigel (Corning) coated plates in LaSR medium according to previously published methods^20, 21^.

### Cardiac differentiation of hPSCs via the GiWi method

When hPSCs maintained on a Matrigel-coated surface achieved confluence, cells were singularized with 0.5 mM EDTA in DPBS at 37 °C for 5 min and then seeded onto a Matrigel-coated cell culture dish at 100,000 cells/cm^2^ in LaSR supplemented with 5 μM Y-27632 (Selleckchem) (day -2) for 24 hours. Cells were then cultured in LaSR without Y-27632, changed daily. Cardiac differentiation of hPSCs was performed according to previously published GiWi method^5, 6, 22^. Briefly, at day 0, cells were treated with 9 µM CHIR99021 (Selleckchem) for 24 hour in RPMI medium supplemented with 2% B27-insulin (Gibco), followed by a change with RPMI + B27-insulin medium at day 1. At day 3, 2 µM Wnt-C59 (Tocris) was added, followed by a medium change with RPMI plus 2% B27 (Gibco) at day 5. Cells were then cultured in RPMI plus 2% B27 with medium change every three days.

### Nucleofection

Two plasmids, XLone-GFP and a pCYL43 plasmid containing a highly active PiggyBac transposase, were used to integrate the transposon into the genome of H9 cells. Two million hPSCs, 2 µg XLone-GFP plasmid and 2 µg pCYL43 plasmid were mixed in 100 µL nucleofection solution and then nucleofected with the B-16 program using a Nucleofector 2b device.

### Transfection using Lipofectamine 3000

A total of 3 μg of plasmid DNA was added to 50 μL DMEM (Gibco) with 5 μL of P3000 (Invitrogen). A second solution of 3.75 μL lipofectamine 3000 (Invitrogen) in 50 μL DMEM was made. Both solutions incubated at room temperature for 5 minutes prior to combination, followed by 10 min incubation at room temperature. The mixture was then pipetted into one well of cells and incubated overnight at 37 °C. The media was changed on the cells the next day.

### Fluorescence-activated cell sorting

Doxycycline was administered to cells at 2000 ng/mL 24 hours prior to sorting. Cells were then washed with DPBS and dissociated into a single cell suspension with 0.5 mM EDTA in DPBS for 15 minutes at 37 °C. Cell suspension was pelleted and re-suspended in DPBS with 0.5% BSA and 5 µM Y27632. Cells were then sorted on a Beckman Coulter MoFlo Astrios and the cell population with highest GFP expression was collected.

### Flow cytometry analysis

To analyze GFP expression, cells were dissociated into single cells with 0.5 mM EDTA in DPBS for 10 min at 37 °C and then added at a 1:2 v/v ratio to DPBS with 0.5% BSA. Data were collected on a Beckman Coulter FC500 flow cytometer and analyzed using FlowJo. FACS gating was based on the corresponding untreated cell control.

### Immunostaining

Cells were fixed with 4% paraformaldehyde for 15 min at room temperature and then immunostained with primary and secondary antibodies (Supplementary Table 1) in DPBS with 0.4% Triton X-100 and 5% non-fat dry milk. Nuclei were stained with Hoechst 33342. A Nikon Ti Eclipse epifluorescence microscope was used for imaging analysis.

## Electronic supplementary material

Supplementary Info
