# Supplementary material for: An all-in-one, Tet-On 3G inducible PiggyBac system for human pluripotent stem cells and derivatives
Source: Sci Rep. 2017 May 8;7:1549. doi: 10.1038/s41598-017-01684-6 (PMC5431539; doi:10.1038/s41598-017-01684-6)
Supplement: Supplementary file 1 — Supplementary Info [file 41598_2017_1684_MOESM1_ESM.pdf]

An all-in-one, Tet-On 3G inducible PiggyBac system for human pluripotent stem cells and derivatives

Lauren N. Randolph[1,3,#], Xiaoping Bao[4,#], Chikai Zhou[5], Xiaojun Lian[1,2,3,*]

[1]Department of Biomedical Engineering, [2]Department of Biology, [3]The Huck Institutes of the Life Sciences, Pennsylvania State University, University Park, PA, 16802, USA
[4]Department of Chemical and Biological Engineering, University of Wisconsin, Madison, WI, 53706, USA
[5]Department of Cell and Molecular Biology, Karolinska Institutet, 17177, Stockholm, Sweden
* Correspondence should be addressed to: Xiaojun Lian (Lian@psu.edu)
# These authors contributed equally to this work.

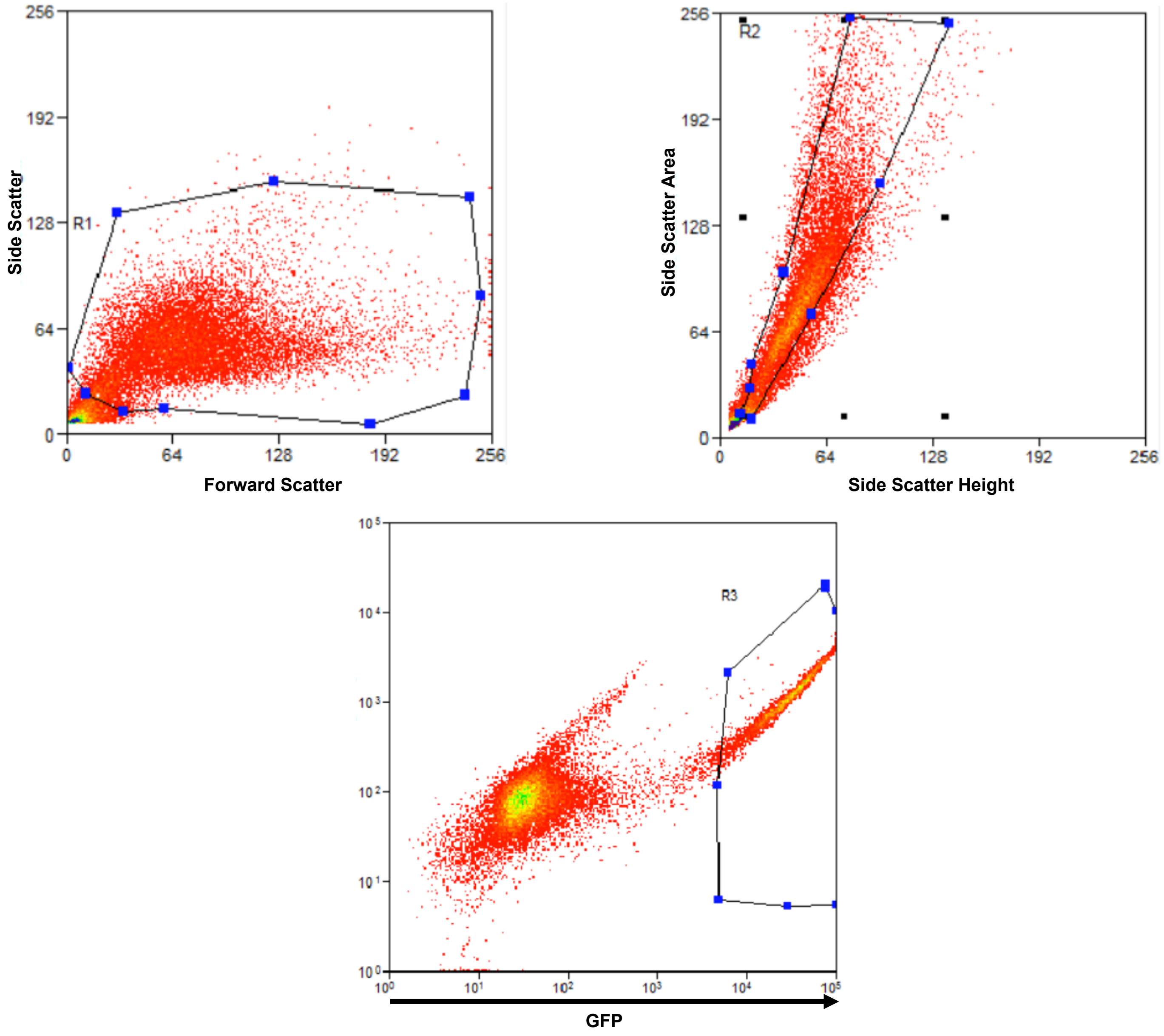

Supp. Fig. 1: FACS sort gating for population with highest GFP expression (R3).

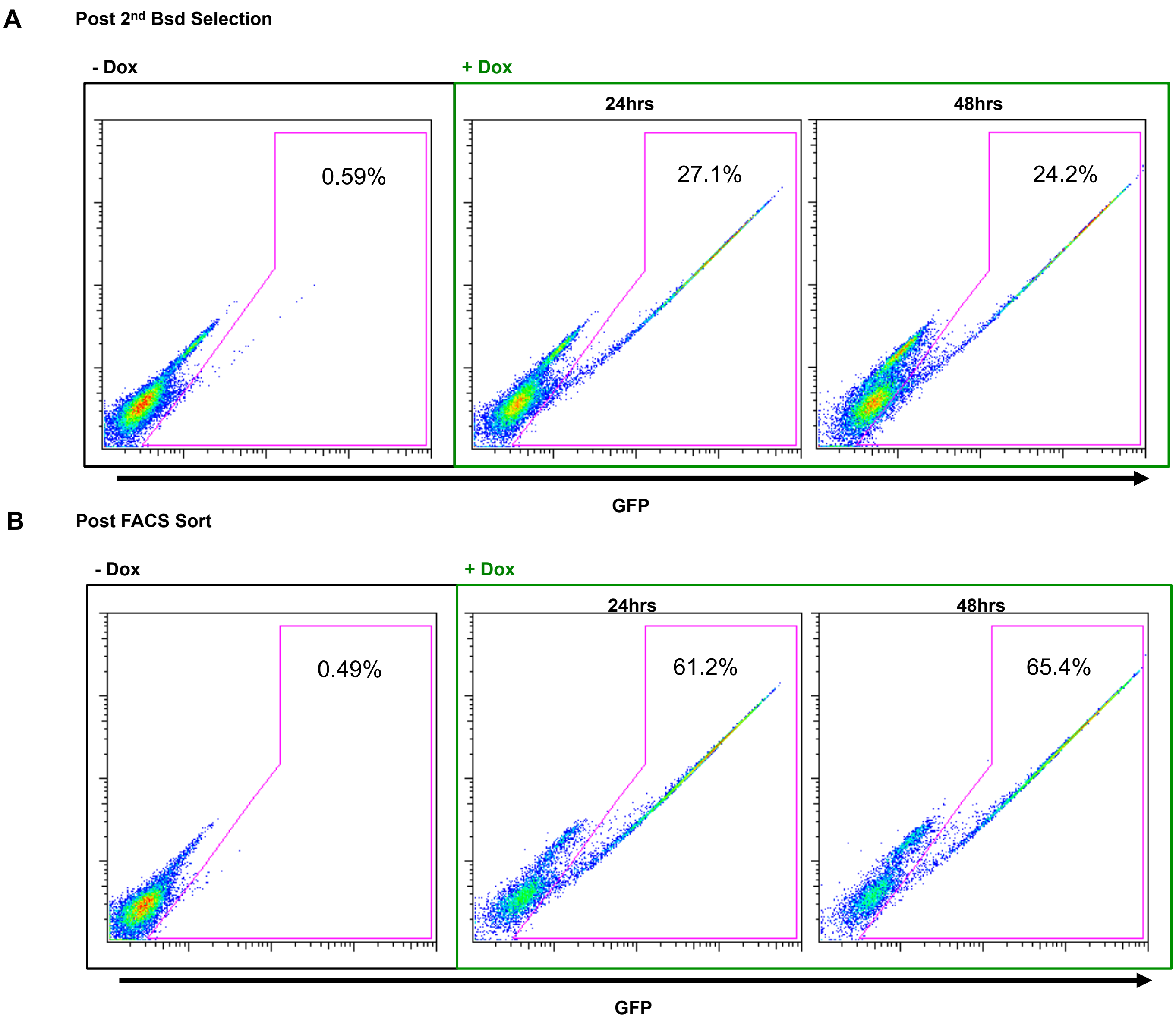

Supp. Fig. 2: Comparison of GFP expression at 24 and 48 hours in cells after A) two rounds of Bsd selection and B) FACS sorting.

Antibody	Source	Application
Oct3/4	Santa Cruz, mouse IgG_{2b}, Clone: C-10, sc-5279	1:100 (IS)
Nanog	Thermo Fisher Scientific, rabbit IgG, PA1-097	1:500 (IS)
cTnT	Lab Vision, mouse IgG1, Clone: 13-11, ms: 295-p1	1:200 (IS)
SSEA-4	DSHB, mouse IgG3, MC-813-70	1:20 (IS)
cTnI	Santa Cruz, rabbit IgG, sc-15368/H-170	1:100 (IS)
Nkx2.5	Santa Cruz, rabbit IgG, sc-14033/H-114	1:75 (IS)
Secondary Antibody	Alexa 555 Goat anti Rb IgG, A-21428	1:1000
Secondary Antibody	Alexa 647 Goat anti Rb IgG, A-21244	1:1000
Secondary Antibody	Alexa 647 Goat anti Ms IgG, A-21235	1:1000

Supp. Table 1: Antibodies used in immunostaining analysis.
